# Identifying symptomatic adverse events using the patient‐reported outcomes version of the common terminology criteria for adverse events in patients with non‐small cell lung cancer with epidermal growth factor receptor exon 20 insertion mutations

**DOI:** 10.1002/cam4.5376

**Published:** 2022-12-30

**Authors:** Yanyan Zhu, Milenka Jean‐Baptiste, William R. Lenderking, Jill A. Bell, Dennis A. Revicki, Huamao M. Lin, Rachael Brake, Bryce B. Reeve

**Affiliations:** ^1^ Millennium Pharmaceuticals, Inc., a wholly owned subsidiary of TakedaPharmaceutical Company Limited Massachusetts Cambridge USA; ^2^ Evidera Inc. Bethesda Maryland USA; ^3^ Evidera Inc. Waltham Massachusetts USA; ^4^ Duke University School of Medicine Durham North Carolina USA

**Keywords:** adverse events, non‐small cell lung cancer, oncology, patient‐reported outcome measures, PRO‐CTCAE, symptoms, tolerability

## Abstract

**Objective:**

Tolerability and safety of treatments are important in oncology trials and should be informed by patient assessments. We identified the most relevant patient‐reported symptomatic adverse events (AEs) to measure in patients with non‐small cell lung cancer (NSCLC) with epidermal growth factor receptor (*EGFR*) exon 20 insertion mutations.

**Methods:**

This study selected relevant symptomatic AEs from 78 AEs available in the Patient‐Reported Outcomes version of the Common Terminology Criteria for Adverse Events (PRO‐CTCAE) measurement system. Initially, symptomatic AEs were selected based on literature and product labeling reviews, and then core sets of symptomatic AEs were identified by patient and clinician interviews. Qualitative and descriptive analyses were performed using the data collected from three iterative rounds of patient interviews.

**Results:**

During concept elicitation interviews involving 29 patients, 12 symptomatic AEs were identified and were then adapted into a 25‐item PRO‐CTCAE form for use in future clinical trials along with commonly used PRO measures. Cognitive interviews showed that the PRO‐CTCAE items were easy to answer and appropriate for assessing the patients' experience with symptomatic AEs. This study also assessed disease symptoms, impacts, and overall patient experience.

**Conclusions:**

The 25‐item PRO‐CTCAE form captures the most relevant symptomatic AEs in this patient population, and it is available for future studies. Baseline characterization of AEs associated with this distinct patient group contributes to our broader knowledge about NSCLC and through platforms like Project Patient Voice will expand our understanding of treatment tolerability and safety for NSCLC. Ultimately, this data collection will help inform decision‐making for patients, caregivers, healthcare providers, and regulators.

## Graphical abstract

This figure shows the multiple steps in the process of selecting, testing, and refining a set of PRO‐CTCAE items specific to patients with NSCLC *EGFR* exon20 insertion mutations. The inputs included literature review, clinical expert opinion, several rounds of patient input and testing, and label review.

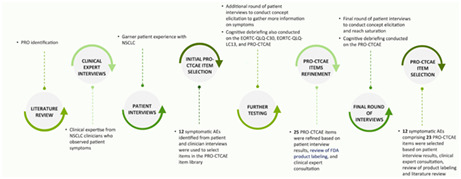

## 1 INTRODUCTION

Patient‐reported data are increasingly recognized as an important source of information about the symptoms, impact, and tolerability of both a disease and its treatments. In oncology, where multiple treatments might be available with similar efficacy profiles, differentiation may be observed in adverse events (AEs). As such, capturing the patient perspective regarding AEs may influence treatment decisions during the course of a patient's treatment journey or inform decision‐making for future patients with a similar diagnosis.

Traditionally, in oncology clinical trials, AEs are graded by clinicians using the National Cancer Institute's (NCI's) Common Terminology Criteria for Adverse Events (CTCAE),^1^ and patient‐reported symptomatic AEs are not always systematically captured. Evidence suggests that patient reports may differ from clinician assessments of safety data, often presenting contrasting data that could influence future treatment decisions.^2^ Moreover, clinicians have been known to underestimate the impact of the given symptoms on patients' lives.^3^

Approximately, 4%–12% of non‐small cell lung cancer (NSCLC) cases with mutations in the epidermal growth factor receptor gene (*EGFR*) harbor exon 20 insertion mutations (Exon 20ins). Given the rarity of these *EGFR* mutations, the identification of this patient population is not well established. At the time of conducting this analysis, there was no specific treatment strategy recommended in European or US guidelines, and patients were generally treated with chemotherapy or *EGFR* tyrosine kinase inhibitors.^4^ Recently, amivantamab and mobocertinib received Breakthrough Therapy Designations from the US FDA for the treatment of patients with metastatic NSCLC with *EGFR* Exon 20ins, whose disease has progressed on or after platinum‐based chemotherapy.^4^, ^5^, ^6^ The evidence on the efficacy and safety of currently available therapies (e.g., nonexon 20‐targeted) has been poor and hence there is a great unmet need for the development of more targeted and safe therapies for this rare subset of the patient population.^4^, ^7^ Nonspecific therapies like platinum‐based chemotherapy are known to adversely impact patients' quality of life,^8^ and documenting this effect contributes to the value potential of developing *EGFR* Exon 20ins‐specific therapies.

The NCI's Patient‐Reported Outcomes version of the CTCAE (PRO‐CTCAE) was developed to capture symptomatic AEs directly from patients.^9^ However, it is broad and encompasses different cancer types, and thus needs to be customized for specific treatments. Despite the advocacy of the US FDA and NCI initiatives, there is no standard use of the PRO‐CTCAE in oncology clinical trials, including NSCLC. Thus, this study was conducted to identify the relevant PRO‐CTCAE items for patients with NSCLC with *EGFR* Exon 20ins that would be relevant for future studies and describes the process undertaken for identifying and validating the relevant concepts. In addition to symptomatic AEs, disease symptoms and impacts were also evaluated. Thus, this paper is also an early report on the disease‐related symptoms and impacts of this not well‐understood patient population.

## 2 METHODS

**FIGURE 1:**
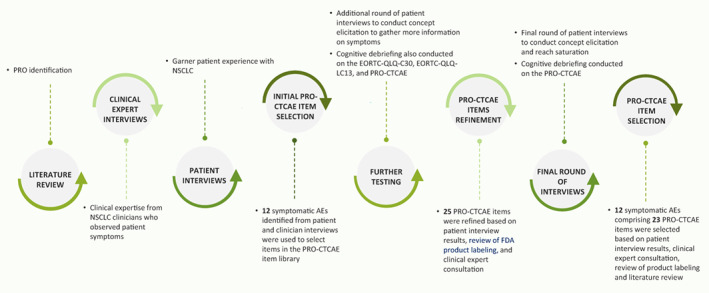
Process undertaken to develop the PRO‐CTCAE‐specific form for patients with NSCLC with *EGFR* Exon 20ins. AE, adverse event; *EGFR*, epidermal growth factor receptor gene; EORTC QLQ‐C30, European Organization for Research and Treatment of Cancer‐Quality of Life Questionnaire; EORCT‐QLQ‐LC13, EORTC QLQ‐Lung Cancer 13; Exon 20ins, exon 20 insertion mutations; FDA, Food and Drug Administration; NSCLC, non‐small‐cell lung cancer; PRO, patient‐reported outcome; PRO‐CTCAE, Patient‐Reported Outcomes version of the Common Terminology Criteria for Adverse Events

To identify the PRO‐CTCAE item set specific for patients with NSCLC with *EGFR* Exon 20ins from literature, product labeling, clinician interviews, and directly from patients' input (including both concept elicitation and cognitive interviews), study steps were implemented iteratively (Figure 1).

### 2.1 Literature and product labeling review and clinician interviews

First, a literature review was conducted to identify patient symptoms and health‐related quality of life (HRQOL) impacts along with relevant patient‐reported measures used in patients with NSCLC with *EGFR* Exon 20ins. A targeted search algorithm outlined the search terms to be used when looking through the EMBASE and PubMed databases for articles related to the use of patient‐reported outcome (PRO) measures and NSCLC in the literature (Appendix S1). Selected abstracts were screened by primary (HA) and secondary (MJB) reviewers who determined whether they were relevant enough to merit a full‐text article review. Data from the full‐text articles reflecting these abstracts were extracted and tabled. The PRO measures identified during this process were also tabled and compared to one another to assess which PRO measures had better concept coverage. A psychometric comparison of selected PRO measures was also done. A product labeling review was also conducted to assess the common AEs among commonly used therapies, including cisplatin and carboplatin, as per the National Comprehensive Cancer Network guidelines.^10^ Both FDA and EMA labeling claim databases were searched. Adverse events for products that used the European Organization for Research and Treatment of Cancer (EORTC) or other PRO measures for lung cancer were selected for further assessment. These AEs were then ranked by frequency of occurrence in the labeling claims and were discussed for further inclusion in the PRO‐CTCAE based on their relevance to treatments for NSCLC with *EGFR* Exon 20ins.

Of these 10 PRO measures identified in the literature review, the EORTC – Quality of Life Questionnaire (EORTC QLQ‐C30) and EORTC QLQ‐Lung Cancer 13 (LC13) had the best concept coverage of symptoms for patients with NSCLC with *EGFR* Exon 20ins. Although several concepts were identified in the literature review, there were limited articles distinguishing between disease‐related symptoms and treatment‐related symptomatic AEs. Thus, subsequent interviews focused on delineating between what patients considered disease‐related symptoms and treatment‐related symptomatic AEs. The PRO‐CTCAE was subsequently adapted to assess the symptomatic AEs during patient interviews.

In addition, six oncology specialists were interviewed to explore NSCLC symptoms and impacts from a clinical perspective and to collect feedback on the preliminary conceptual disease model developed based on the literature review. Each information source informed the development of the patient interview guides, as well as preliminary and final PRO‐CTCAE item selection.

### 2.2 Sample for patient interviews

Study participants were eligible if they had NSCLC with *EGFR* Exon 20ins, were ≥18 years old at the time of recruitment, read and spoke English, and had experienced symptoms within the previous 30 days. Inclusion criteria were slightly tailored in different rounds of patient interviews to achieve specific study objectives. For example, to evaluate symptomatic AEs associated with commonly used therapies, in the third round of interviews, patients were also required to have received platinum‐based chemotherapy within the past 2 years. Patients throughout the USA were recruited through a mix of patient advocacy organizations and a clinical recruitment vendor. Interviews were conducted between October 2017 and May 2019. Advarra IRB (formerly Chesapeake) exempted this study from institutional review board oversight. All patients provided written informed consent.

### 2.3 PRO measures

The adult version of NCI's PRO‐CTCAE includes a library of 124 items that assess 78 symptomatic AEs from the CTCAE.^9^ The items have previously undergone extensive qualitative and quantitative evaluation to support their validity and reliability.^9^, ^11^, ^12^ For each symptomatic AE (e.g., headache), there are up to three questions related to key symptom attributes, including the symptom frequency, severity, and interference with daily activities. Each question uses a 7‐day recall with either a 5‐point response scale assessing frequency, severity, or interference with daily activities, or a binary response scale assessing the presence or absence of a symptomatic AE.

In addition to the PRO‐CTCAE, the EORTC QLQ‐C30 and the EORTC QLQ‐LC13 were completed by participants to assess symptoms, impacts, and overall experience associated with NSCLC. The EORTC QLQ‐C30 is a 30‐item questionnaire that assesses the HRQOL of patients with cancer. It has been widely used in oncology trials and translated into over 80 languages.^13^ The questionnaire contains responses ranging from 1 (“Not at all”) to 4 (“Very much”). The EORTC QLQ‐LC13 is a supplement to the EORTC QLQ‐C30 and consists of 13 items that focus on symptoms and daily activities in patients with lung cancer.^14^

### 2.4 Approach for patient interviews

This was a qualitative study involving telephone‐based, one‐on‐one interviews with patients with NSCLC with *EGFR* Exon 20ins conducted by qualified researchers using a semi‐structured interview guide (Appendix S2). Interviews incorporated approaches from both concept elicitation^15^ and cognitive interviewing.^16^ Interviews were up to 90 min long, recorded, and transcribed. Interviews were conducted over the phone.

Three rounds of interviews were conducted with sample sizes greater than seven in each round. For cognitive interviewing, sample sizes of 6–12 individuals are typically recommended, with multiple rounds to evaluate changes and feedback from previous rounds.^17^ The PRO‐CTCAE and other measures assessed during cognitive interviews were mailed to participants in a sealed envelope, and they were instructed to open it at the start of the interview.

Round 1 interviews focused on assessing patient symptoms, impacts, and the ability of the commonly used PRO measures (i.e., EORTC QLQ‐C30 and EORTC QLQ‐LC13) to adequately assess the experiences of patients, especially related to treatment‐related symptomatic AEs. During this interview round, patients spontaneously endorsed both disease‐related symptoms and treatment‐related symptomatic AEs, which were experienced *after* treatment initiation. The symptomatic AEs identified during Round 1 interviews led to the development of the initial item subset selected from the PRO‐CTCAE measurement system.

The PRO‐CTCAE items selected from Round 1 were evaluated using cognitive interviewing methods during Rounds 2 and 3. During Round 2 interviews, in addition to patient input for selected symptomatic AEs from the PRO‐CTCAE, we also received input from six clinicians and reviewed product labeling from pemetrexed/cisplatin or pemetrexed/carboplatin that is one of the commonly used therapies. The Round 3 interviews included those patients receiving a platinum‐based chemotherapy regimen.

A semi‐structured interview guide was developed for each interview round and interviews were audio‐recorded. Our process was consistent with recommendations by Trask et al. on selecting items from the PRO‐CTCAE.^18^

### 2.5 Analyses

All quantitative data from the interviews were collected using DataFax (DF/Net Research, Inc.). Descriptive statistics were calculated using SAS 9.4 (SAS Institute Inc.).

A concept frequency grid was developed to enumerate symptoms endorsed across all three rounds of patient interviews. To be included in the final PRO‐CTCAE version for patients with NSCLC with *EGFR* Exon 20ins, one of the following three conditions had to be met: (i) symptomatic AEs endorsed by patients in all three rounds of interviews, (ii) endorsed by patients in at least two rounds of interviews with endorsement by clinicians or identified as an AE from product labeling search, or (iii) endorsed by patients in one round of interviews but also have clinician endorsement, be identified as an AE from the product labeling search, and appear as an AE in previous trials of the targeted treatment of interest.^19^, ^20^ Saturation was defined as the point in the interviews when no additional concepts were mentioned.

The analysis was done using ATLAS.ti version 7.5.11 (ATLAS.ti Scientific Software Development GmbH, Berlin)^21^ to identify codes and count symptoms. Symptoms were assumed to be disease‐related if they were reported prior to treatment initiation and potentially considered symptomatic AEs if reported to occur posttreatment onset, although it was recognized that some might be disease‐related symptoms.

Additional details of the qualitative interview process are provided in Appendix S2.

## 3 RESULTS

### 3.1 Sociodemographic and clinical characteristic results

Across all three rounds of interviews, a total of 29 patients with NSCLC with *EGFR* Exon 20ins were recruited (Table 1). Overall, the median (range) patient age was 53.0 (33.0–83.0) years. Approximately half of the patients were female (51.7%) and the majority were White (*n* = 18; 62.1%). On average, patients had been diagnosed with NSCLC for 1.8 years (standard deviation = 2.0 years). Most patients did not have brain metastases (*n* = 21; 72.4%) and their current medications included anticancer agents (79.3%), pain medications/corticosteroids (37.9%), and antibiotics (31.0%). The majority of patients had received chemotherapy as their cancer treatment (82.8%), followed by radiation (48.3%) and targeted therapy (44.8%) (Table 1).

**TABLE 1:** Sociodemographic and clinical characteristics across all three interview rounds

Characteristic	Round 1 (*n* = 9)	Round 2 (*n* = 7)	Round 3 (*n* = 13)	Total (*N* = 29)
Age (years)				
Mean ± SD	56.56 (12.78)	54.86 (5.49)	50.31 (8.00)	53.34 (9.42)
Median (range)	55.0 (42.00–83.00)	56.0 (45.00–62.00)	51.0 (33.00–63.00)	53.0 (33.00–83.00)
Female, n (%)	5 (55.6)	3 (42.9)	7 (53.8)	15 (51.7)
Racial background, *n* (%)^a^ ^,^ ^b^				
White	9 (100.0)	5 (71.4)	4 (30.8)	18 (62.1)
Black or African American	0 (0.0)	0 (0.0)	5 (38.5)	5 (17.2)
Asian	0 (0.0)	2 (28.6)	1 (7.7)	3 (10.3)
Other	0 (0.0)	0 (0.0)	1 (7.7)	1 (3.4)
Missing	0 (0.0)	0 (0.0)	2 (15.4)	2 (6.9)
Ethnicity, *n* (%)				
Hispanic or Latino	0 (0.0)	0 (0.0)	2 (15.4)	2 (6.9)
Non‐Hispanic or Latino	9 (100.0)	7 (100.0)	9 (69.2)	25 (86.2)
Missing	0 (0.0)	0 (0.0)	2 (15.4)	2 (6.9)
Employment status, *n* (%)^a^				
Employed, full‐ and part‐time	5 (55.6)	5 (71.5)	1 (7.7)	11 (37.9)
Homemaker	0 (0.0)	0 (0.0)	1 (7.7)	1 (3.4)
Unemployed	0 (0.0)	1 (14.3)	3 (23.1)	4 (13.8)
Retired	2 (22.2)	0 (0.0)	2 (15.4)	4 (13.8)
Disabled	3 (33.3)	1 (14.3)	5 (38.5)	9 (31.0)
Missing	0 (0.0)	0 (0.0)	1 (7.7)	1 (3.4)
Education, *n* (%)^c^				
Secondary/high school	1 (11.1)	0 (0.0)	1 (7.7)	2 (6.9)
Associate degree, technical or trade school	1 (11.1)	0 (0.0)	1 (7.7)	2 (6.9)
Some college	0 (0.0)	1 (14.3)	3 (23.1)	4 (13.8)
College degree	0 (0.0)	4 (57.1)	7 (53.8)	11 (37.9)
Postgraduate degree	7 (77.8)	2 (28.6)	0 (0.0)	9 (31.0)
Missing	0 (0.0)	0 (0.0)	1 (7.7)	1 (3.4)
Time since diagnosis (years)				
Mean ± SD	2.2 (2.0)	3.4 (2.6)	0.7 (0.6)	1.8 (2.0)
Median (range)	1.6 (0–6)	2.7 (1–7)	0.6 (0–2)	1.1 (0–7)
Brain metastases, *n* (%)				
Missing	0 (0.0)	0 (0.0)	1 (7.7)	1 (3.4)
Yes	5 (55.6)	2 (28.6)	0 (0.0)	7 (24.1)
No	4 (44.4)	5 (71.4)	12 (92.3)	21 (72.4)
Current medications, *n* (%)^a^				
Pain medications/corticosteroids	5 (55.6)	4 (57.1)	2 (15.4)	11 (37.9)
Anticancer agents (e.g., chemotherapy)	7 (77.8)	5 (71.4)	11 (84.6)	23 (79.3)
Nutritional support	1 (11.1)	3 (42.9)	1 (7.7)	5 (17.2)
Antibiotics	6 (66.7)	3 (42.9)	0 (0.0)	9 (31.0)
Correction of metabolic syndrome disorder	0 (0.0)	1 (14.3)	0 (0.0)	1 (3.4)
Other^d^	4 (44.4)	1 (14.3)	0 (0.0)	5 (17.2)
No medications	1 (11.1)	1 (14.3)	0 (0.0)	2 (6.9)
Cancer treatments received, *n* (%)^a^				
Surgery^e^	4 (44.4)	2 (28.6)	1 (7.7)	7 (24.1)
Radiation	7 (77.8)	6 (85.7)	1 (7.7)	14 (48.3)
Chemotherapy	8 (88.9)	7 (100.0)	9 (69.2)	24 (82.8)
Prophylactic brain radiation	2 (22.2)	0 (0.0)	0 (0.0)	2 (6.9)
Targeted therapy^f^	9 (100.0)	4 (57.1)	0 (0.0)	13 (44.8)
Photodynamic therapy	0 (0.0)	0 (0.0)	0 (0.0)	0 (0.0)
Radiofrequency ablation	0 (0.0)	0 (0.0)	0 (0.0)	0 (0.0)
No treatments	0 (0.0)	0 (0.0)	0 (0.0)	0 (0.0)
Other^g^	3 (33.3)	0 (0.0)	0 (0.0)	3 (10.3)

Abbreviation: PRN, as needed; SD, standard deviation.

^a^ Not mutually exclusive, therefore may not sum to 100%. Cancer treatments received may include past treatments as well as current chemotherapy.

^b^ Other race included: Hispanic (as reported by the patient; *n* = 1).

^**c**^ Other education included: Law school (*n* = 1) and radiology technologist, RTR (*n* = 1).

^d^ Other current medications included: Florastor (*n* = 1), Poziotinib, Savasya (blood thinner), Imodium (PRN), Ativan (PRN) (*n* = 1), radiation (*n* = 1), Tylenol, blood thinner Lovenox (*n* = 1), immunotherapy (*n* = 2).

^e^ Surgeries specified included: prostate, 20 years ago (also Cyberknife surgery for brain tumors 2017) (*n* = 1), r‐lung wedge‐resection (*n* = 1), segmentectomy (*n* = 1), endoscopic cranial biopsy (*n* = 1), lung (*n* = 1), node removal (*n* = 1), prophylactic pleurodesis (*n* = 1).

^f^ Targeted therapies (provided by patients) listed included atatinib’ (*n* = 1), atatinib (*n* = 1), Avastin (*n* = 2), carboplatin (*n* = 1), Keytruda (*n* = 1), necitumumab (*n* = 1), nivolumab (*n* = 1), pembrolizumab (*n* = 1), Portrazza (*n* = 1), poziotinib (*n* = 8), Tagrisso (*n* = 1), Tarceva (*n* = 2), Tecentiq (*n* = 1).

^g^ Other cancer treatments received included immunotherapy (*n* = 3) and Zometa for bones (*n* = 1).

### 3.2 Patient‐reported NSCLC treatment‐related symptomatic AEs and PRO‐CTCAE refinement results

Across all three interview rounds, AEs were defined as those that patients largely reported experiencing *after* treatment initiation. Symptomatic AEs identified for possible PRO‐CTCAE inclusion by patients, clinicians, and other sources are summarized in Table 2, and representative quotations are provided in Figure S1.

**TABLE 2:** Symptomatic AEs identified for possible PRO‐CTCAE inclusion by patients, clinicians, and other sources by frequency of patient endorsement

Symptomatic AEs	Round 1 (*n* = 9)	Round 2 (*n* = 7)	Round 3 (*n* = 13)	Mean	Identified from product labeling review	Core symptoms identified for solid tumors^26^	NCI Symptom Management and HRQOL Steering Committee^18^, ^27^	Selected for final PRO‐CTCAE
Nausea^a^	33%	100%	54%	62%	X	X	X	X
Diarrhea^a^	78%	43%	39%	53%	X	X	X	X
Vomiting^a^	22%	43%	8%	24%	X	X		X
Lack of appetite^b^	56%	0%	62%	39%	–	X	X	X
Sores in the mouth or tongue	78%	0%	15%	31%	X	–	–	X
Weight loss^a^	56%	14%	0%	23%	–	–	–	–
Rash^a^ ^,^ ^b^	78%	0%	0%	26%	X			X
Constipation	44%	0%	23%	22%	X	X	X	X
Dry skin^a^ ^,^ ^b^	33%	29%	0%	21%	–	–	–	X
Cracked nails	0%	57%	0%	19%	–	–	–	–
Itchy skin^a^	0%	57%	0%	19%	–	–	–	–
Hair loss	11%	43%	0%	18%	–	–	–	–
Fatigue^a^	33%	14%	15%	21%	X	X	X	X
General pain	11%	14%	15%	13%	X	X	X	X
Tingling^b^	22%	0%	0%	7%	–	X		X
Insomnia/Disturbed sleep	0%	14%	23%	12%	–	X	X	–
Weakness	11%	0%	23%	11%	–	–	–	–
Shivering/shaking chills^b^	11%	14%	0%	8%	X	–	–	X
Dizziness	17%	0%	8%	8%	–	–	–	–
Arm or leg swelling	0%	0%	23%	8%	X	–	–	–
Cough	0%	0%	15%	5%	–	–	–	–
Abdominal pain^a^	0%	14%	0%	5%	–	–	–	–
Trouble swallowing^a^	11%	0%	0%	4%	–	–	–	–
Pounding or racing heartbeat	0%	0%	8%	3%	X	–	–	–
Aching muscles	0%	0%	8%	3%	X	–	–	–
Aching joints	0%	0%	8%	3%	X	–	–	–
Voice changes	0%	0%	8%	3%	X	–	–	–
Watery eyes	0%	0%	8%	3%	X	–	–	–
Nosebleeds	0%	0%	8%	3%	X	–	–	–
Shortness of breath	0%	0%	0%	0%	–	X	X	–
Easily bruised	0%	0%	0%	0%	X	–	–	–
Cognitive problems^c^	0%	0%	0%	0%	–	–	X	–
Depression/Sadness^c^	0%	0%	0%	0%	–	X	X	–
Anxiety/Distress	0%	0%	0%	0%	–	X	X	–
Sensory neuropathy^c^	0%	0%	0%	0%	–	–	X	–
Dry mouth	0%	0%	0%	0%	–	X	–	–

Abbreviations: AE, adverse event; HRQOL, health‐related quality of life; NCI, National Cancer Institute; PRO‐CTCAE, Patient‐Reported Outcomes version of the Common Terminology Criteria for Adverse Events.

^a^ From previous trials of targeted treatment of interest.^19^, ^20^

^b^ Clinician endorsed.

^c^ These symptomatic AEs were only reported by the NCI Symptom Management and HRQOL Steering Committee.

During Round 1 interviews, patients reported a total of 17 symptomatic AEs (Table 2). Based on the results of the concept elicitation interviews, the number of symptomatic AEs was narrowed to seven and included the following: lack of appetite, rash, diarrhea, dry skin, nausea, vomiting, and fatigue. Symptomatic AEs were narrowed down based on a few factors, including (i) the proportion of patients endorsing symptomatic AEs; (ii) triangulation with clinician interviews and literature review and what patients were saying; and (iii) AEs listed in the PRO‐CTCAE library as well as in commonly used PRO measures.

Although not reported by patients during Round 1 interviews, five symptomatic AEs were added due to their possible relationship with NSCLC or its treatments (acne, abdominal pain, shortness of breath, cough, and trouble swallowing) based on commonly known symptomatic AEs and internal data (data not shown). Thus, by the end of Round 1 interviews, 12 symptomatic AEs from the PRO‐CTCAE were considered for further testing with patients.

During Round 2 interviews, patients identified 13 symptomatic AEs. The most frequently reported AEs were nausea, itchy skin, cracked nails, diarrhea, vomiting, hair loss, and dry skin (Table 2). Of the AEs reported by patients during Round 2 interviews, 69% overlapped with the AEs identified during Round 1 interviews (nausea, vomiting, diarrhea, dry skin, weight loss, fatigue, hair loss, general pain, and shivering/shaking chills), whereas 31% were entirely new (pain in the abdomen, itchy skin, cracked nails, and insomnia). Additionally, product labeling review of AEs reported in carboplatin or cisplatin trials yielded eight additional concepts. These concepts (arm or leg swelling, easily bruised, pounding or racing heartbeat, aching muscles, aching joints, voice changes, nosebleeds, and watery eyes) were tested during Round 3 interviews (Table 2). Finally, seven items that were identified during Round 1 interviews but not reported by patients during Round 2 interviews included the following: sores in the mouth or tongue, rash, lack of appetite, constipation, tingling, trouble swallowing, and dizziness. With the exception of weight loss, hair loss, and insomnia, the following 25 symptomatic AEs were considered for further testing with patients during Round 3 interviews: diarrhea, dry skin, nausea, vomiting, fatigue, shivering/shaking chills, general pain, abdominal pain, itchy skin, cracked nails, rash, sores in the mouth or tongue, lack of appetite, constipation, tingling, trouble swallowing, dizziness, arm or leg swelling, easily bruised, pounding or racing heartbeat, aching muscles, aching joints, voice changes, nosebleeds, and watery eyes. Weight loss, in particular, was not included, as it was considered an objective sign. Furthermore, it was not an item included in the NCI core set.

These 25 symptomatic AEs were evaluated in a third and final round of concept elicitation interviews to confirm the appropriateness of the PRO‐CTCAE items proposed for the patient population of interest, including patient experience with platinum‐based chemotherapy. During Round 3 interviews, a total of 12 symptomatic AEs were identified by patients. The most frequently reported AEs were decreased/lack of appetite, nausea, diarrhea, weakness, insomnia, and constipation.

Across all three rounds of interviews, patients reported that they had experienced 26 different symptomatic AEs. Of these, the most frequently reported symptoms were experienced predominantly *after* treatment initiation and included the following: nausea, diarrhea, lack of appetite, sores in the mouth or tongue, rash, vomiting, weight loss, constipation, and dry skin. One additional symptomatic AE, fatigue, was also considered important based on CTCAE results from previous clinical trials in a similar patient population.^19^, ^20^ This confirmed what we noted during patient interviews.^19^ Similarly, tingling in the hands or feet and shivering/shaking chills were also considered important because they were endorsed by clinical experts, despite not having been frequently endorsed by the patients. Finally, although the pain was not one of the most frequently endorsed symptomatic AEs, it was endorsed by patients across all three rounds of interviews and was thus considered important to assess in future studies. At the end of all three rounds of concept elicitation interviews, 12 symptomatic AEs comprising 25 items that were relevant for patients with NSCLC with *EGFR* Exon 20ins were selected from the PRO‐CTCAE (Table 2).

### 3.3 Cognitive debriefing of selected PRO‐CTCAE items

Patients were cognitively debriefed about the selected PRO‐CTCAE items during Round 2 interviews and were then asked to individually describe what each item on the study‐specific PRO‐CTCAE meant to them. Most patients showed adequate comprehension of each item. Additionally, patients generally had a favorable impression of the PRO‐CTCAE. When asked about their overall impression of the PRO‐CTCAE, patients generally responded as “thorough,” “seemed relevant,” “easy to understand,” and “very clear.” When probed on their understanding of individual items on the PRO‐CTCAE, patients demonstrated good comprehension. Patients also indicated that they felt 12 concepts represented in the 25‐item PRO‐CTCAE form reflected their experiences with NSCLC with *EGFR* Exon 20ins and that it was not missing any AEs relevant to their experience.

### 3.4 Additional patient‐reported NSCLC concepts

In addition to reporting symptomatic AEs, patients also described disease‐specific symptoms and impacts. Across all three rounds of interviews, symptoms were defined as those which patients largely reported experiencing prior to treatment initiation. The top symptoms reported across all three rounds of interviews included the following: shortness of breath, fatigue/tiredness, chest pain, cough, and appetite loss (Figure S2). These represent the core symptoms typically associated with NSCLC that are endorsed by clinical experts (e.g., cough, dyspnea, chest pain). Although appetite loss was predominantly reported as a symptomatic AE, some patients also reported it as a symptom experienced *prior* to treatment initiation. Half of the clinical experts interviewed also endorsed appetite loss as a symptom. Thus, it has been reported as both a symptom and a symptomatic AE.

When asked how NSCLC impacted their lives, patients frequently mentioned impacts that fell into seven categories (emotional, social functioning, role functioning, physical functioning, work/occupational capacity, cognitive functioning, and financial) (Appendix S3).

## 4 DISCUSSION

This study identified important symptomatic AEs experienced by patients with NSCLC with *EGFR* Exon 20ins. By following a robust process, including using multiple information sources, conducting the research in several rounds, adapting patient interviews and study samples, and clinical experts' input, we identified a core set of 12 symptomatic AEs that are directly relevant to NSCLC with *EGFR* Exon 20ins and its treatments, notwithstanding their relevance to other forms of NSCLC.

During the cognitive debriefing of selected PRO‐CTCAE items, most patients indicated that each item was relevant to their experiences of the disease, clear, and understandable. Although patients mentioned that none of the key symptomatic AEs was missing, fatigue was considered an important symptomatic AE based on findings from previous clinical trials as well as our observations during patient interviews,^19^, ^20^ whereas tingling in the hands or feet and shivering/shaking chills were added to the list based on clinical experts' input. Most symptoms endorsed by clinical experts, which were core symptoms and typically associated with NSCLC disease, were endorsed by most patients as well. The majority of the patients were impacted emotionally because of their disease condition.

Over 50% of items identified in this study are commonly reported by patients with NSCLC as well. Results from a cross‐sectional survey involving patients with metastatic NSCLC on immunotherapy or chemo‐immunotherapy showed that fatigue, rash, lack of appetite, constipation, dry skin, diarrhea, and nausea were the most prevalent symptoms (at least moderate or occasional) experienced in the last week on the PRO‐CTCAE.^22^ Since most patients had received different cancer treatments (e.g., chemotherapy, targeted therapy), items included in the PRO‐CTCAE may be useful in identifying/capturing symptomatic AEs related to a range of treatments.

### 4.1 Study limitations

Given the small sample size of this study, by seeking interviews with clinical experts and a review of CTCAE data from clinical trials in early development, we sought to minimize the risk of missing an important but uncommon side effect. This approach also helped in capturing any symptoms that would not have been reported by patients, as they were being asked about their symptom experience for a new treatment that they had not received before. Recall bias is another potential concern with collecting data on symptoms and AEs, as it may not be clear whether the impact was by virtue of not having been interviewed while on treatment or the inability of patients to differentiate whether the AE experienced is treatment‐ or disease‐related, especially if they were treated with multiple chemotherapy lines. Targeted therapy with *EGFR* Exon 20ins inhibitors is still evolving and new treatments may bring in new AEs, which could be a limitation of the current study and/or a topic for prospective studies. Additionally, this study may not have accounted for the impact that co‐morbidities may have had on patients and the symptomatic AEs, symptoms, and impacts reported.

The focus on patients with NSCLC with *EGFR* Exon 20ins is both a strength and a limitation of our study. This is a rare subgroup of NSCLC, making it difficult to recruit more patients, and it is important to have qualitative data on uncommon subgroups of patients when developing assessments for them, when possible. Previous research has shown that *EGFR* Exon 20ins have similar clinical characteristics to common *EGFR* mutations.^23^ Similarly, when looking at the patient experience, it was our conclusion that this subgroup did not differ symptomatically in meaningful ways from patients with other NSCLC forms, but this conclusion was necessarily based on a small sample.

The last limitation of this research is that not all symptomatic AEs might be identified in advance when studying an experimental drug going into Phase 2 or Phase 3. This would mean that the PRO‐CTCAE might still miss some symptomatic AEs associated with the treatment. On the other hand, it is unnecessarily burdensome to give the entire PRO‐CTCAE item library to all patients. To address this concern, this study used available information on treatments for this patient population, including EGFR tyrosine kinase inhibitors and chemotherapy, in order to cast a slightly broader net of relevant items. The PRO‐CTCAE for certain treatments of certain cancer types can be adjusted after Phase 2 or Phase 3 studies and after additional AEs have emerged. Furthermore, the PRO‐CTCAE subset for treatment can be developed for Phase 2 studies that are likely to be placebo‐controlled, thus making it easier to distinguish treatment‐related events.

### 4.2 Clinical implications

Our efforts should be distinguished from those of others to identify a core set of lung cancer symptoms; for example, the 4‐item Pulmonary Symptom Index,^24^ and the 7‐item NSCLC Symptom Assessment Questionnaire,^25^ because although similar qualitative methods were used to establish content validity, our focus was on symptomatic AEs that could result from treatment rather than symptoms of the disease. Researchers using Eastern Cooperative Oncology Group (ECOG) data proposed a core set of 13 disease‐ and treatment‐related symptoms which were tested in solid tumors, including breast, prostate, colon/rectum, and lung.^26^ The NCI Symptom Management and HRQOL Steering Committee also identified a core set of 12 symptoms, which are presented in Table 2.^18^, ^27^ Although there was overlap between eight of the treatment‐related symptoms identified from the ECOG data^26^ and the symptomatic AEs identified in this study, we identified four additional symptomatic AEs (sores in mouth or tongue, rash, dry skin, cold in hands or feet) as we focused specifically on the *EGFR* Exon 20ins form of the NSCLC disease. During Round 2 interviews, two patients with NSCLC with *EGFR* Exon 20ins were also reported as being anaplastic lymphoma kinase positive. For analysis and reporting purposes, they were classified as having *EGFR* with Exon 20ins. Further, our research did not include five of the treatment‐related symptoms from the ECOG set (disturbed sleep, dry mouth, distress, shortness of breath, and sadness). Regarding the NCI set, six symptomatic AEs overlapped with our list, we identified six symptoms that were not on the list, and their list included five symptomatic AEs that were not on our list as the NCI's focus was across all types of cancers. This finding supports the validity of our approach by the substantial convergence between the three studies but also emphasizes the value of conducting research targeted to a specific disease and set of treatments. Moreover, the study findings highlight the importance of understanding the patient perspective on treatment‐related symptomatic AEs experienced by this rare subgroup of NSCLC. The EORTC measures were not developed to assess treatment‐related symptomatic AEs specifically, so supplementing those measures or other PRO efficacy measures with a treatment‐specific PRO‐CTCAE can help to provide a more comprehensive perspective on treatment. Furthermore, treatment‐specific PRO‐CTCAE items, when used in a clinical trial setting, can contribute to burgeoning knowledge around AEs in the field of oncology through platforms, such as the FDA's Project Patient Voice.^28^

## 5 CONCLUSIONS

This study has identified a set of symptomatic AEs and disease symptoms and impacts associated specifically with a patient population identified to have NSCLC with *EGFR* Exon 20ins and establishes one of the first baseline assessments as to our understanding of this emerging patient population. The robust process adapted for this study allowed us to identify a set of 12 most relevant symptomatic AEs from the PRO‐CTCAE's item library. This item set showed greater than 50% agreement with a core set of symptoms previously proposed for solid tumors, yet also identified additional symptoms that are of importance to this specific patient population. Similarly, compared to the NCI Symptom Management and HRQOL Steering Committee's 12 core symptoms to be used in oncology trials, there was greater than a 50% overlap in items selected. This work can now enable future clinical research with targeted therapies focused on this unique and tough‐to‐treat patient subset while also more broadly contributing to the assessment of tolerability and safety of treatment for NSCLC relevant to the patient experience.

## AUTHOR CONTRIBUTIONS

**Yanyan Zhu:** Conceptualization (equal); data curation (equal); formal analysis (equal); writing – original draft (equal); writing – review and editing (equal). **Milenka Jean‐Baptiste:** Conceptualization (equal); data curation (equal); formal analysis (equal); writing – original draft (equal); writing – review and editing (equal). **William R. Lenderking:** Conceptualization (equal); data curation (equal); formal analysis (equal); writing – original draft (equal); writing – review and editing (equal). **Jill A. Bell:** Conceptualization (equal); data curation (equal); formal analysis (equal); writing – original draft (equal); writing – review and editing (equal). **Dennis A. Revicki:** Conceptualization (equal); data curation (equal); formal analysis (equal); writing – original draft (equal); writing – review and editing (equal). **Huamao M. Lin:** Formal analysis (equal); writing – original draft (equal); writing – review and editing (equal). **Rachael Brake:** Conceptualization (equal); data curation (equal); formal analysis (equal); writing – original draft (equal); writing – review and editing (equal). **Bryce B. Reeve:** Conceptualization (equal); writing – original draft (equal); writing – review and editing (equal).

## FUNDING INFORMATION

Evidera, an outcomes research specialty firm, received funding from Takeda in order to carry out this work.

## CONFLICT OF INTEREST

YZ and JAB were former employees of Takeda when this research was conducted and are current employees of AstraZeneca. HML and RB are current employees of Takeda. WRL is a full‐time employee of Evidera, DAR was a former employee of Evidera (now deceased), and MJB is a part‐time employee of Evidera, which received funding from Takeda to carry out this research. BBR served as a paid consultant for Takeda.

## ETHICS APPROVAL AND CONSENT TO PARTICIPATE

The study was reviewed and exempted by Advarra IRB (formerly Chesapeake) using the Department of Health and Human Service regulations found at 45 CFR 46.101(b) (2) (Pro00023602), and all patients gave written permission to participate.

## Supporting information


Figure S1

Figure S2

Appendix S1

Appendix S2

Appendix S3

## Data Availability

The data that support the findings of this study are available from the corresponding author upon reasonable request.
