# Supplementary material for: Identifying symptomatic adverse events using the patient‐reported outcomes version of the common terminology criteria for adverse events in patients with non‐small cell lung cancer with epidermal growth factor receptor exon 20 insertion mutations
Source: Cancer Med. 2022 Dec 30;12(5):5494–505. doi: 10.1002/cam4.5376 (PMC10028096; doi:10.1002/cam4.5376)
Supplement: Supplementary file 1 — Figure S1 [file CAM4-12-5494-s004.pdf]

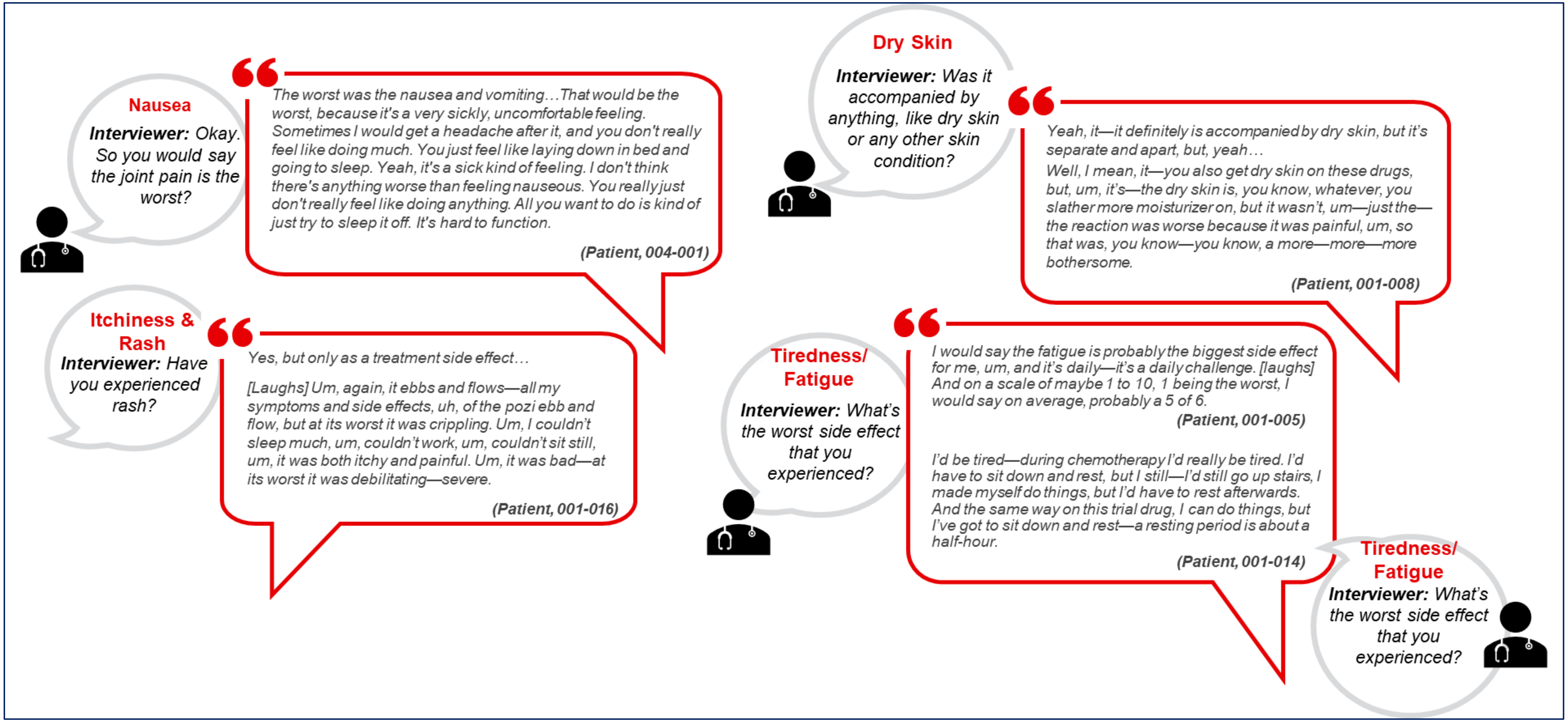
Nausea
Interviewer: Okay. So you would say the joint pain is the worst?
The worst was the nausea and vomiting…That would be the worst, because it's a very sickly, uncomfortable feeling. Sometimes I would get a headache after it, and you don't really feel like doing much. You just feel like laying down in bed and going to sleep. Yeah, it's a sick kind of feeling. I don't think there's anything worse than feeling nauseous. You really just don't really feel like doing anything. All you want to do is kind of just try to sleep it off. It's hard to function.
(Patient, 004-001)
Itchiness & Rash
Interviewer: Have you experienced rash?
Yes, but only as a treatment side effect…
[Laughs] Um, again, it ebbs and flows—all my symptoms and side effects, uh, of the pozi ebb and flow, but at its worst it was crippling. Um, I couldn't sleep much, um, couldn't work, um, couldn't sit still, um, it was both itchy and painful. Um, it was bad—at its worst it was debilitating—severe.
(Patient, 001-016)
Dry Skin
Interviewer: Was it accompanied by anything, like dry skin or any other skin condition?
Yeah, it—it definitely is accompanied by dry skin, but it's separate and apart, but, yeah…
Well, I mean, it—you also get dry skin on these drugs, but, um, it's—the dry skin is, you know, whatever, you slather more moisturizer on, but it wasn't, um—just the—the reaction was worse because it was painful, um, so that was, you know—you know, a more—more—more bothersome.
(Patient, 001-008)
Tiredness/ Fatigue
Interviewer: What's the worst side effect that you experienced?
I would say the fatigue is probably the biggest side effect for me, um, and it's daily—it's a daily challenge. [laughs] And on a scale of maybe 1 to 10, 1 being the worst, I would say on average, probably a 5 of 6.
(Patient, 001-005)
I'd be tired—during chemotherapy I'd really be tired. I'd have to sit down and rest, but I still—I'd still go up stairs, I made myself do things, but I'd have to rest afterwards. And the same way on this trial drug, I can do things, but I've got to sit down and rest—a resting period is about a half-hour.
(Patient, 001-014)
Tiredness/ Fatigue
Interviewer: What's the worst side effect that you experienced?
