# Supplementary figures and images for: Identifying symptomatic adverse events using the patient‐reported outcomes version of the common terminology criteria for adverse events in patients with non‐small cell lung cancer with epidermal growth factor receptor exon 20 insertion mutations
Source: Cancer Med. 2022 Dec 30;12(5):5494–505. doi: 10.1002/cam4.5376 (PMC10028096; doi:10.1002/cam4.5376)

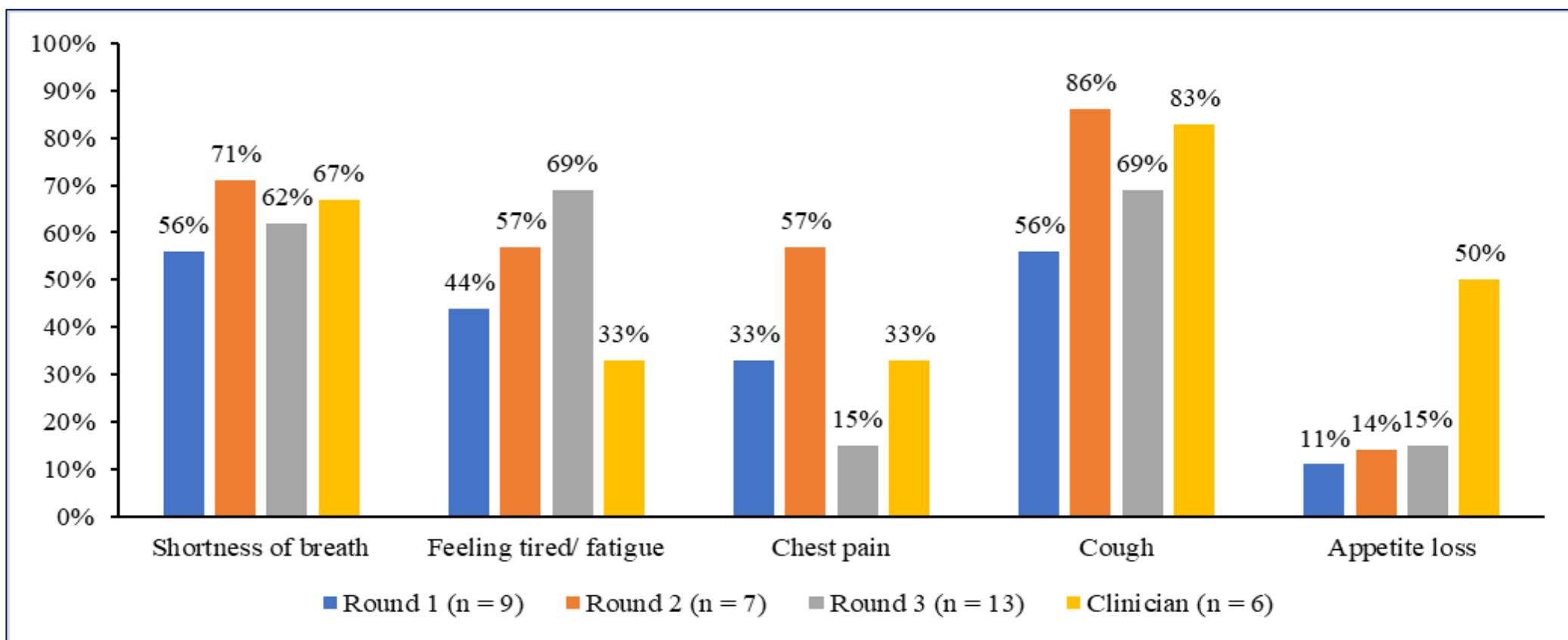

Supplement: Supplementary file 2 — Figure S2 [file CAM4-12-5494-s001.pdf]
