# Supplementary material for: Identifying symptomatic adverse events using the patient‐reported outcomes version of the common terminology criteria for adverse events in patients with non‐small cell lung cancer with epidermal growth factor receptor exon 20 insertion mutations
Source: Cancer Med. 2022 Dec 30;12(5):5494–505. doi: 10.1002/cam4.5376 (PMC10028096; doi:10.1002/cam4.5376)
Supplement: Supplementary file 3 — Appendix S1 [file CAM4-12-5494-s003.docx]

Search Terms and Strategy Worksheet

EMBASE Search Algorithm

| Search Number | Search Terms | Search Yield |
| --- | --- | --- |
| #1 | 'non small cell lung cancer'/exp OR 'lung non small cell cancer'/exp OR 'lung alveolus cell carcinoma'/exp OR ('lung':ab,ti AND 'adenocarcinoma':ab,ti) OR ('lung':ab,ti AND 'large cell carcinoma':ab,ti) | 130126 |
| #2 | ('pro measure*':ab,ti OR 'patient reported outcome*':ab,ti OR ('treatment outcome'/exp/mj AND patient:ab,ti) OR ('self reported':ab,ti AND outcome*:ab,ti) OR ('self evaluation'/exp/mj AND outcome*:ab,ti) AND (measure*:ab,ti OR assess*:ab,ti) OR (‘self evaluation’:ab,ti AND outcome*:ab,ti) AND (measure*:ab,ti OR assess*:ab,ti) OR ('patient reported' AND outcome*:ab,ti AND measure*:ab,ti) OR proms OR pro OR 'patient* NEAR/2 reported') AND ('interview'/exp OR interview*:ab,ti OR 'questionnaire'/exp OR questionnaire*:ab,ti OR survey*:ab,ti OR 'instrument'/exp OR instrument*:ab,ti OR 'rating scale'/exp OR scale*:ab,ti OR 'measurement'/exp OR assessment*:ab,ti OR index*:ab,ti OR diar*:ab,ti OR inventor*:ab,ti OR score*:ab,ti OR profile:ab,ti OR profiles:ab,ti OR 'named inventories, questionnaires and rating scales'/exp OR (measur* NEAR/2 (behav* OR function*)):ab,ti OR ‘social functioning’:ab,ti AND 'quality of life'/exp OR 'quality of life':ti,ab OR 'hrql':ab,ti OR hrqol:ab,ti OR qol:ab,ti OR ‘life quality’:ti,ab OR ‘well-being’:ti,ab OR qualitative:ti,ab OR 'qualitative research'/exp OR 'wellbeing'/exp OR 'value of life':ab,ti OR 'patient satisfaction'/exp OR (patient* NEAR/2 satisfaction):ab,ti OR 'self report'/exp OR (self NEAR/1 report*):ab,ti OR 'patient preference'/exp OR (patient* NEAR/2 preference*):ab,ti OR (patient* NEAR/1 assess*):ab,ti OR ‘self evaluations’:ti,ab OR (patient* NEAR/2 rating):ab,ti OR (patient* NEAR/2 rated):ab,ti OR ‘self-completed’:ti,ab OR 'self-administered':ti,ab OR (self NEAR/1 assessment*):ab,ti OR ‘self-rated’:ti,ab OR 'patient based outcome':ti,ab OR 'self evaluation'/exp OR experience*:ab,ti OR ‘patient completed’:ab,ti) | 44756 |
| #3 | #1 AND #2 | 251 |

PubMed Algorithm

| Search Number | Search Terms | Search Yield |
| --- | --- | --- |
| #1 | "Carcinoma, Non-Small-Cell Lung"[Mesh] OR “lung alveolus cell carcinoma”[tiab] OR (“lung”[tiab] AND “adenocarcinoma”[tiab]) OR (“lung”[tiab] AND “large cell carcinoma”[tiab]) | 62516 |
| #2 | (“pro measure*”[tiab] OR “patient reported outcome*”[tiab] OR "Patient outcome assessment"[MESH] OR "self reported"[Text Word] AND outcome*[Text Word] OR "Self-Assessment"[majr] AND outcome[tiab] AND (measure*[tiab] OR assess*[tiab]) OR "self assessment"[tw] AND outcome*[tw] OR “self evaluation”[tiab] AND outcome*[tiab] AND (measure*[tiab] OR assess*[tiab]) OR ("patient reported"[tw] AND outcome*[tw] OR PROMS[tw] OR PRO[tw] OR Patient satisfaction[mesh]) OR ("satisfaction"[tiab] AND (patient[tiab] OR treatment[tiab])) OR "patient compliance"[mesh] OR ((treatment[tiab] OR patient[tiab]) AND compliance[tiab]) OR Adherence*[tiab]) OR Attitude[tiab] OR "Patient centered"[tiab]) OR "Patient experience"[tiab] OR "Patient driven" OR "intervention"[tiab] OR "self-monitor" OR "patient-provider communication"[tiab]) AND ("Interviews as Topic"[Mesh] OR interview[tiab] OR interviews[tiab] OR interviewed[tiab] OR interviewing[tiab] OR "questionnaires"[MeSH Terms] OR questionnaire[TIAB] OR questionnaires[TIAB] OR survey[TIAB] OR surveys[TIAB] OR instrument[TIAB] OR instruments[TIAB] OR scale[TIAB] OR scales[TIAB] OR measure[TIAB] OR measures[TIAB] OR measurement[TIAB] OR measurements[TIAB] OR assessment[TIAB] OR assessments[TIAB] OR index[TIAB] OR diary[TIAB] OR diaries[TIAB] OR inventory[TIAB] OR inventories[TIAB] OR score[TIAB] OR scores[TIAB] OR profile[tiab] OR profiles[tiab]) OR (behav*[tiab] AND measur*[tiab]) OR (function*[tiab] AND measur*[tiab]) OR “social functioning”[tiab]) AND ("quality of life"[MESH] OR "quality of life"[TIAB] OR HRQL[TIAB] OR HRQOL[TIAB] OR QOL[TIAB] OR well-being[tiab] OR qualitative[TIAB] OR "Qualitative Research"[Mesh] OR “life quality”[tiab] OR “value of life”[tiab] OR "Value of Life"[Mesh] OR "Patient Satisfaction"[Mesh] OR "patients satisfaction"[TIAB] OR "patient reported"[TIAB] OR "Self Report"[Mesh] OR "self report"[TIAB] OR “self reporting”[TIAB] OR "Patient Preference"[Mesh] OR “patient preference”[TIAB] OR “patient preferences”[TIAB] OR “patients preference”[TIAB] OR “patients preferences”[TIAB] OR “patient assessment”[TIAB] OR “patient assessments”[TIAB] OR “patient assessed”[TIAB] OR “self evaluations”[TIAB] OR “patient rating”[TIAB] OR “patient ratings”[TIAB] OR “patient rated”[TIAB] OR “patients rating”[TIAB] OR “patients ratings”[TIAB] OR “patients rated”[TIAB] OR “self-completed”[TIAB] OR “self-administered”[TIAB] OR “patient completed”[TIAB] OR “self assessments”[TIAB] OR “patient based outcome”[TIAB] OR "Self-Evaluation Programs"[Mesh] OR experience[TIAB] OR experiences[TIAB]) | 202005 |
| #3 | #1 AND #2 | 337 |

SUMMARY

| Activity Description | # of Abstracts Yielded |
| --- | --- |
| Embase Search | 251 |
| Pub Med Search | 337 |
| Total Abstract Yield | 588 |
| Total Abstract Yield when duplicates are removed | 548 |
