# Supplementary material for: Identifying symptomatic adverse events using the patient‐reported outcomes version of the common terminology criteria for adverse events in patients with non‐small cell lung cancer with epidermal growth factor receptor exon 20 insertion mutations
Source: Cancer Med. 2022 Dec 30;12(5):5494–505. doi: 10.1002/cam4.5376 (PMC10028096; doi:10.1002/cam4.5376)
Supplement: Supplementary file 4 — Appendix S2 [file CAM4-12-5494-s005.docx]

**Additional details of the qualitative interviews**

| Qualitative interviews |
| --- |
| Study organizational structure |
| - The study was conducted by the sponsor (Takeda) in collaboration with a contract research organization (Evidera) |
| Interview Description   - Concept elicitation interviews were conducted to identify treatment- and disease-related symptoms, particularly relevant to NSCLC *EGFR* Exon 20ins and impacts on HRQOL. Cognitive interviews were conducted to evaluate understandability and content validity of select PRO-CTCAE items   Researchers |
| - Two female health outcomes research professionals (HA and MJB) conducted interviews. Each interviewer have master’s degrees and have been trained in qualitative data collection and this study purpose |
| Process |
| - Patient organizations and a clinical recruitment vendor screened participants and provided the researchers with contact information of interested and eligible patients - One of the researchers (HA) then contacted potential participants by telephone to schedule telephonic interviews - Audio files were transcribed verbatim by Gordon Transcripts, Inc., a transcription vendor - Transcripts were not returned to patients for comment or correction |
| Analysis |
| - An ATLAS.ti coding dictionary was developed based on the themes and concepts that emerged during patient interviews - Coding schemes were reviewed and revised based on findings from patient interviews by HA and MJB - Training was provided to ensure that the coder understood the meaning and purpose of each code and to promote consistency in coding - Several transcripts were coded by two researchers (HA and MJB) and reviewed for accuracy and consistency by another researcher (LR). Discrepancies were discussed and reconciled |

Abbreviations: EGFR = epidermal growth factor receptor; Exon 20ins = exon 20 insertion mutations; HRQOL = health-related quality of life; NSCLC = non-small cell lung cancer; PRO-CTCAE = Patient-Reported Outcomes version of the Common Terminology Criteria for Adverse Events.
