# Supplementary material for: Identifying symptomatic adverse events using the patient‐reported outcomes version of the common terminology criteria for adverse events in patients with non‐small cell lung cancer with epidermal growth factor receptor exon 20 insertion mutations
Source: Cancer Med. 2022 Dec 30;12(5):5494–505. doi: 10.1002/cam4.5376 (PMC10028096; doi:10.1002/cam4.5376)
Supplement: Supplementary file 5 — Appendix S3 [file CAM4-12-5494-s002.docx]

**Most frequent patient-reported impacts (N = 29)**

| **Type of Impact** | **Percentage reporting**  ***N* (%)** |
| --- | --- |
| Emotional | 26 (90) |
| Worry/Anxiety | 14 (48) |
| Stress | 7 (24) |
| Depression | 6 (21) |
| Social functioning | 21 (72) |
| Family life | 11 (38) |
| Ability to carry out errands | 8 (28) |
| Role functioning | 20 (69) |
| Ability to carry out hobbies | 14 (48) |
| Ability to carry out household chores | 13 (45) |
| Physical functioning | 14 (48) |
| Walk up or down stairs without difficulty | 12 (41) |
| Diminished ability to carry heavy objects | 8 (28) |
| Work/Occupational capacity | 9 (31) |
| Cognitive functioning | 7 (24) |
| Ability to concentrate | 5 (17) |
| Issues with memory/trouble remembering things | 4 (14) |
| Financial | 5 (17) |
| Treatment cost | 2 (7) |
| Impact on income | 2 (7) |
| Ability to pay bills | 1 (3) |
